# Multiple structure alignment with msTALI

**DOI:** 10.1186/1471-2105-13-105

**Published:** 2012-05-20

**Authors:** Paul Shealy, Homayoun Valafar

**Affiliations:** 1 Department of Computer Science and Engineering, University of South Carolina, 315 Main Street, Columbia, SC 29208, USA

## Abstract

**Background:**

Multiple structure alignments have received increasing attention in recent years as an alternative to multiple sequence alignments. Although multiple structure alignment algorithms can potentially be applied to a number of problems, they have primarily been used for protein core identification. A method that is capable of solving a variety of problems using structure comparison is still absent. Here we introduce a program *msTALI* for aligning multiple protein structures. Our algorithm uses several informative features to guide its alignments: torsion angles, backbone C^α^ atom positions, secondary structure, residue type, surface accessibility, and properties of nearby atoms. The algorithm allows the user to weight the types of information used to generate the alignment, which expands its utility to a wide variety of problems.

**Results:**

msTALI exhibits competitive results on 824 families from the Homstrad and SABmark databases when compared to Matt and Mustang. We also demonstrate success at building a database of protein cores using 341 randomly selected CATH domains and highlight the contribution of msTALI compared to the CATH classifications. Finally, we present an example applying msTALI to the problem of detecting hinges in a protein undergoing rigid-body motion.

**Conclusions:**

msTALI is an effective algorithm for multiple structure alignment. In addition to its performance on standard comparison databases, it utilizes clear, informative features, allowing further customization for domain-specific applications. The C++ source code for msTALI is available for Linux on the web at http://ifestos.cse.sc.edu/mstali.

## Background

Multiple sequence alignment techniques have proven useful for identifying related residues from a set of homologous sequences [1-4]. These algorithms provide residue-residue correspondences between sequences in an attempt to identify regions with similar structural, functional, or evolutionary relationships. While useful, these techniques often falter when presented with a set of sequences of low identity. This problem may be overcome using structural information, when available, because structures typically diverge at a rate far lower than sequences [1,5,6]. Furthermore, the rich information available from structures has proven useful in constructing a meaningful alignment because of the structural conservation required for a protein to retain its function [7,8]. For proteins with similar functions, including those with low sequence identity, there is often a common *core*, which frequently contains residues required for proper folding and correct function [9].

Previous approaches to structure alignment can be roughly divided into three groups by their structural representation. 3D methods score structures under rigid-body superposition without allowing flexibility of structures about any hinges. Many recent 3D methods use backbone atom positions but allow flexibility at some backbone positions during alignment. Mustang [10] uses a combination of short fragment alignments and contact maps. POSA [11] and Matt [12] both use aligned fragment pair chaining methods. These approaches all belong to the class of sequential aligners. 2D methods describe structures by their tertiary interactions, such as distance matrices in DALI [13] or contact maps in TOPOFIT [14]. 1D methods reduce each residue to a vector of relevant properties and apply fast string algorithms. CLEMAPS [15] uses conformational letters - discretized conformational states of protein fragments. Vorometric [16] uses Voroni tessellations to determine the residue’s environment.

YAKUSA [17] uses α and τ angles, while 3D-BLAST [18] uses κ and α angles. Other researchers have used backbone φ and ψ torsion angles [19], while TALI [20] incorporates torsion angles and sequence information and Lesk [21] uses torsion angles and a reduced residue representation. Most 1D methods are designed for fast database searching and have not compared favorably with 3D methods [15], with a limited ability to detect structural relationships between proteins [22]. This is due to the fact that most methods of structure linearization fail to capture the necessary information that encapsulates the full structure of a protein.

The poor relative performance of 1D methods is unfortunate because these approaches allow leveraging a wide variety of existing string algorithms. We introduce an algorithm *msTALI* (multiple structure torsion angle alignment) that is a hybrid 1D-3D method. It initially treats all structures in a linear fashion to identify an initial alignment and proceeds to refine the alignment using a 3D scoring criterion. This approach yields an algorithm that is efficient enough for large-scale analyses, yet is still competitive with state-of-the-art 3D methods at aligning homologous structures. This is possible due, in part, to the inclusion of additional features from each residue not included in any previous work. We consider torsion angles, backbone C^α^ atom positions, secondary structure designation of each residue, residue type, surface accessibility, and properties of nearby atoms. Torsion angles are a useful feature because they allow, to a reasonable approximation, a complete reconstruction of the protein structure in linear time compared to a more common method of contact- maps. It is for this reason that a previous version of msTALI, *TALI*[20]*,* relied on torsion angles along with sequence information. However, it is only through inclusion of the additional features that msTALI is able to meaningfully discriminate between local substructures that would otherwise appear identical. The core computational engine for msTALI, which is implemented in C++, and web version of this software can be accessed through the web at: http://ifestos.cse.sc.edu/mstali This approach to structure alignment yields three major benefits. The first is that the algorithm proves very competitive compared to existing methods for comparison of homologous structures, and we provide results to that effect using established databases and comparison metrics. The second benefit is that this algorithm can be customized to a large number of problems. A configurable multiple structure alignment program has a wide variety of potential applications, including core extraction [7], structural phylogeny, active site identification [23], or construction of threading templates for structure prediction [7,24]. However, different algorithms are typically required in order to address different applications of structure alignment. Customization of msTALI for different applications is easily possible due to the inclusion of a variety of relevant biochemical and biophysical features. We illustrate the flexibility of this approach with an example of hinge detection. Furthermore, we envision that this framework will create avenues for novel applications of multiple structure alignments such as characterizing the transmembrane regions of membrane proteins with distinctive patterns of hydrophobicity. The third benefit is that msTALI has the potential for modifications in order to take full advantage of existing string manipulation techniques such as BLAST [25]. Extension of msTALI in this manner can facilitate its deployment as a structure search technique in application to large databases such as the entire PDB.

We consider three problems in this manuscript in the interest of brevity, and we perform a detailed comparison with current 3D algorithms. The first is an example of using msTALI to locate hinges on a structure undergoing rigid-body motion. The second is the *common core identification* problem, where the algorithm accepts *n* proteins and identifies a structural core that is common among all of them. We define a common core as a set of residues which can be superimposed, through rigid-body rotation and translation, with low backbone RMSD (as implemented in molecular visulization tools). Finally, we consider the issue of *core structural phylogeny*. This requires taking a set of highly diverse structures and dividing them into sets of structures, each containing a common core. This task requires a structure comparison score that accurately distinguishes between closely related and distantly related structures. It is for this reason that some algorithms may be advantageous in identifying homologous proteins but perform poorly in reconstruction of evolutionary relations, especially distantly related relations. Furthermore, some structures may have some similarities, and yet they may not share a common core. For example, two proteins may have the same secondary structural elements that are arranged differently in space. We show that the msTALI score satisfies both requirements.

## Results and discussion

### Rigid-body motion detection

One immediate application of msTALI is analysis of rigid-body motion [26]. These motions may be critical to the function of a protein and are of significant interest in pharmaceutical investigations. The first step in understanding the specific nature of an allosteric transformation is locating the exact points of conformational changes that facilitate a rigid-body motion. These studies require aligning multiple structures in various allosteric conformations to arrive at a consensus regarding the critical “hinge” points. While existing tools are capable of producing such alignments over the conserved regions, they fail to provide additional information regarding the conservation of the mobile region. We illustrate this point using an example of three structures of DNA polymerase I from *Thermus aquaticus.* This protein has three domains: a palm, fingers, and the thumb. The structure undergoes two primary allosteric changes [27], denoted *MI* and *MII*. *MI* closes the fingers around DNA, repositioning the O helix and burying ddCTP. *MII* occurs after *MI* and affects the thumb domain, bringing helices H1 and H2 closer to the DNA. Two rotations create *MI*: a 6° rotation of helices N (residues 638–647), O (residues 658–670), O1 (residues 674-679), and O2 (residues 686–699), and a second rotation of helices N and O. Two rotations create *MII*: a rotation of the thumb domain of 17°, and a second rotation of 12° of the H1 (residues 487–495) and H2 (residues 515–521) helices.

The previous analysis [27] identifies the approximate regions of motion, but not the exact residues involved. We analyzed three previously reported structures (1KTQ, 2KTQ, and 3KTQ) of this protein simultaneously with msTALI to detect the exact points of motion and compared our results to those from STAMP [28] and Matt [12]. The three known structures represent three instances of structural characterization of this protein with X-ray crystallography in various allosteric conformations. The 6° rotation is a subtle one that is not expected to stand out from the background noise, but the other three should. Our analysis of these structures with msTALI utilized only torsion angles (msTALI parameters shown in Additional file 1: Table S4). We identified points of motion using the final conservation score of the torsion angles. We computed the mean score, and all residues with scores at least three standard deviations below the mean were considered possible points of motion.

Tables 1, 2 and 3 illustrate the alignment results of STAMP, Matt and msTALI of the three structures 1KTQ, 2KTQ and 3KTQ respectively. In the interest of brevity, only the relevant portions of the final alignments are shown in these tables. As expected, information obtained from the STAMP and Matt alignments (Tables 1 and 2, the complete alignments shown in Additional file 1: Tables S1 and S2) is limited to an approximate location of some structural disagreement among the three structures. Alignments of STAMP and Matt provide no additional information, which necessitates a complete manual investigation.

**Table 1:** The STAMP alignment of three structures of DNA polymerase I in different conformations

	**480**	**490**	**500**	**510**	**520**		
1KTQ	GHPFNLNSRDQLERVLFDELGLPAISTS—A-----AV-LE--A---LREA-HP						
2KTQ	GHPFNLNSRDQLERVLFDELGLP----AIGKTEKTGKRSTSAAVLEALREAHP						(a)
3KTQ	GHPFNLNSRDQLERVLFDELGLP----AIGKTEKTGKRSTSAAVLEALREAHP						
			-H1-----			-H2---		
	640	650	660	670	680	690		
1KTQ	EGRDIHTETASWMFGVPREAVD-PL--M--R---------RAAKTINFG-VLYGMSAHRLSQEL-AIPYEEAQAFIERYFQS							
2KTQ	EGRDIHTETA----S-----D--PL--M--R---------RAAKTINFG-VLYGMSAHRLSQEL-AIPYEEAQAFIERYFQS						(b)	
3KTQ	EGRDIHTET-------------ASWMFGVPREAVDPLMRRAA-KTINFGVL-YGMSAHRLSQELAIP-YEEAQAFIERYFQS							
	----N-----	----O-------------------	--O1--	--O2----------				
	710			740				
1KTQ	TLEEGRRRGYVETLFGRRRYVPDLEARVKSVREAAERMAFNMPVQGTAADLMKLAMVKLF							
2KTQ	TLEEGRRRGYVETLFGRRRYVPDLEARVKSVREAAERMAFNMPVQGTAADLMKLAMVKLF						(c)	
3KTQ	TLEEGRRRGYVETLFGRRRYVPDLEARVKSVREAAERMAFNMPVQGTAADLMKLAMVKLF							
	-P------	-10	-11	-------Q------------------------				

**Table 2:** The MATT alignment of three structures of DNA polymerase I indifferent conformations

	**480**	**490**	**500**	**510**	**520**	
1KTQ	GHPFNLNSRDQLERVLFDELGLPAIGKTEKTGKRS-------TSAA----VLEALREAH					
2KTQ	GHPFNLNSRDQLERVLFDELGLPA-----------IGKTEKTGKRSTSAAVLEALREAH					(a)
3KTQ	GHPFNLNSRDQLERVLFDELGLPA-----------IGKTEKTGKRSTSAAVLEALREAH					
	-H1-----		-H2---			
640	650	660	670	680	690	
1KTQ	EGRDIHTETASWMFGVPREAV---------------D-----PLMRRAAKTINFGVLYGMSAHRLSQELAIPYEEAQAFIERYFQS					
2KTQ	EGRDIHTETAS----------WMFGVPREAV-----D-----PLMRRAAKTINFGVLYGMSAHRLSQELAIPYEEAQAFIERYFQS					(b)
3KTQ	EGRDIHTETAS--------------------WMFGVPREAVDPLMRRAAKTINFGVLYGMSAHRLSQELAIPYEEAQAFIERYFQS					
	----N-----		----O-------	--O1--	--O2----------	
	710		740			
1KTQ	TLEEGRRRGYVETLFGRRRYVPDLEARVKSVREAAERMAFNMPVQGTAADLMKLAMVKLF					
2KTQ	TLEEGRRRGYVETLFGRRRYVPDLEARVKSVREAAERMAFNMPVQGTAADLMKLAMVKLF					(c)
3KTQ	TLEEGRRRGYVETLFGRRRYVPDLEARVKSVREAAERMAFNMPVQGTAADLMKLAMVKLF					
	-P------	-10	-11	-------Q------------------------		

**Table 3:** The msTALI alignment of three structures of DNA polymerase I in different conformations

	**480**	**490**	**500**	**510**	**520**		
1KTQ	GHPFNLNSRDQLERVLFDELGLPAI---------STSAAVLEALREAHP						
2KTQ	GHPFNLNSRDQLERVLFDELGLPAIGKTEKTGKRSTSAAVLEALREAHP						(a)
3KTQ	GHPFNLNSRDQLERVLFDELGLPAIGKTEKTGKRSTSAAVLEALREAHP						
	-H1-----			-H2---			
	640	650	660	670	680	690	
1KTQ	EGRDIHTETASWMFGVPREAVDPLMRRAAKTINFGVLYGMSAHRLSQELAIPYEEAQAFIERYFQS						
2KTQ	-GRDIHTETAS----------DPLMRRAAKTINFGVLYGMSAHRLSQELAIPYEEAQAFIERYFQS						(b)
3KTQ	EGRDIHTETASWMFGVPREAVDPLMRRAAKTINFGVLYGMSAHRLSQELAIPYEEAQAFIERYFQS						
----N-----	----O-------				--O1--	--O2----------	
	740						
1KTQ	TLEEGRRRGYVETLFGRRRYVPDLEARVKSVREAAERMAFNMPVQGTAADLMKLAMVKLF						
2KTQ	TLEEGRRRGYVETLFGRRRYVPDLEARVKSVREAAERMAFNMPVQGTAADLMKLAMVKLF						(c)
3KTQ	TLEEGRRRGYVETLFGRRRYVPDLEARVKSVREAAERMAFNMPVQGTAADLMKLAMVKLF						
	-P------	-10	-11	-------Q------------------------			

Low-scoring residues are highlighted in yellow. Secondary structures are annotated below the alignment.

**Figure 1:**
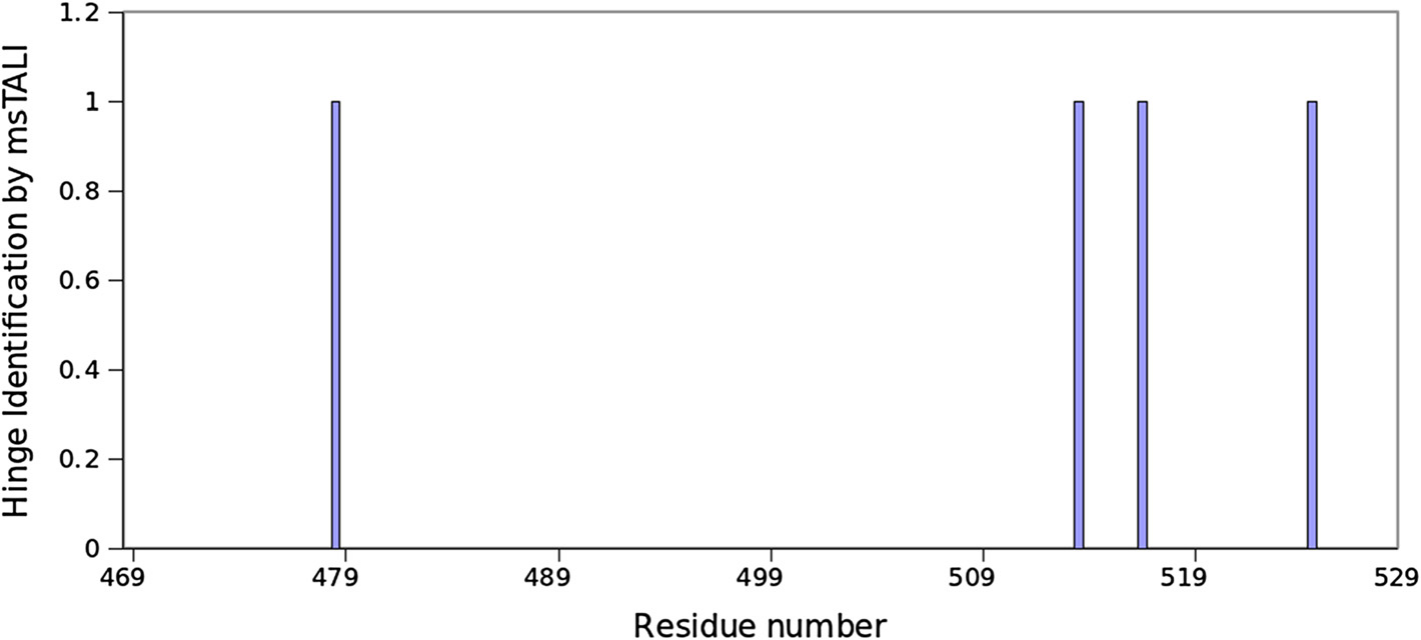
**Scoring profile of msTALI for residues 469–529 in application to the proteins 1KTQ, 2KTQ and 3KTQ.** The four residues displayed in this figure correspond to residues that exhibit individual matching scores that are outside of mean scores of all residues by 3σ. These four residues constitute the hinge regions**.**

**Figure 2:**
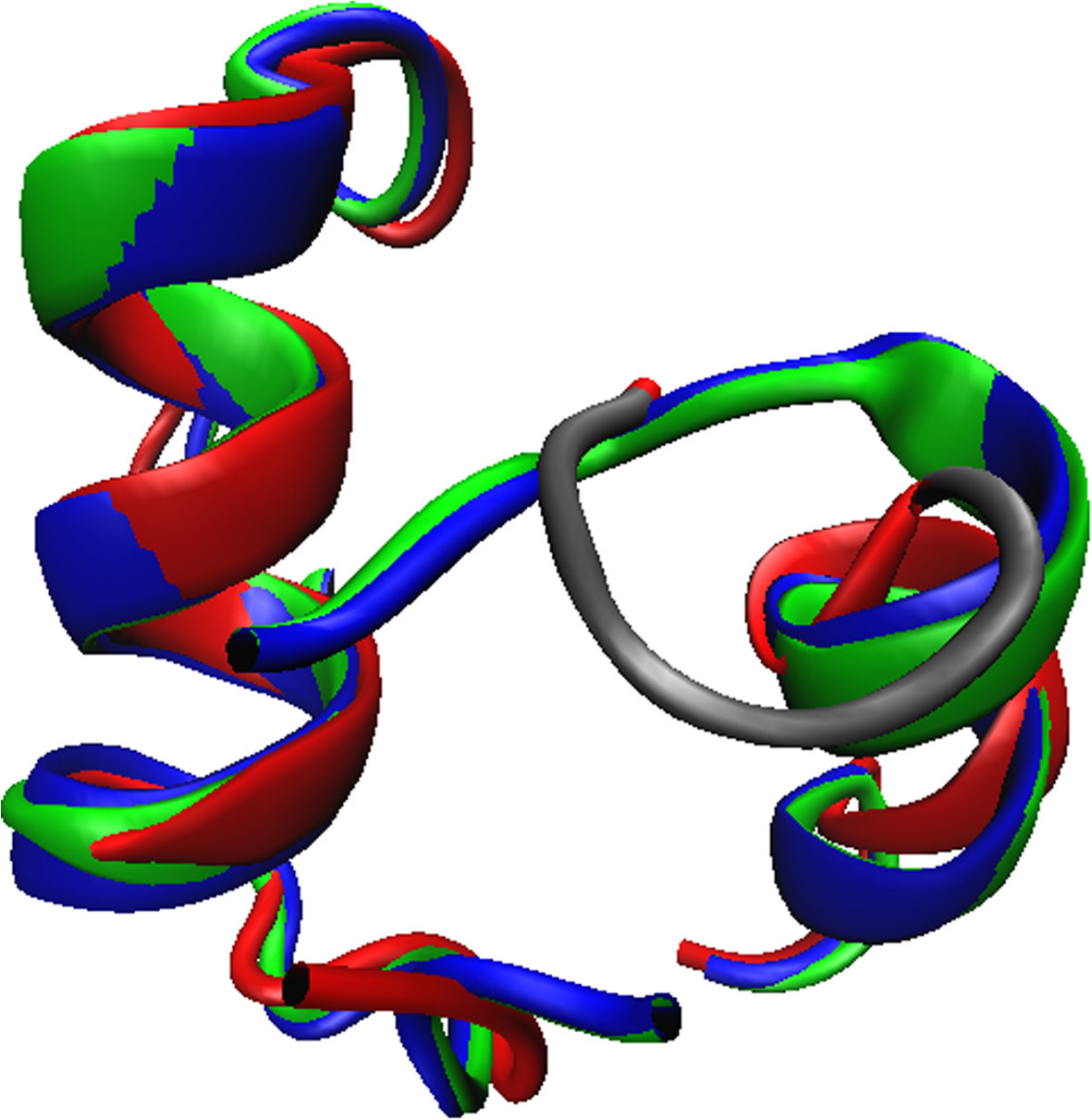
**A superposition of three zinc finger domains, residues 479–525. **1KTQ is shown in red, 2KTQ is green, 3KTQ is blue. An additional hinge region, residues 514–517, is shown in gray**.**

In contrast, results of msTALI alignment are far more informative as illustrated in Table 3 (the complete alignment is in Additional file 1: Table S3). Based on results shown in this table, all structurally conserved regions across all three structures are identified. In addition to the conserved structural regions, the hinge points that accommodate conformational changes can be identified by observing per-residue score that is provided by msTALI. The per-residue score provides information regarding the structural conservation of each residue using the final alignment information. Residues with significant deviations in their scores (more than 3σ in this report) can be identified as exact location of structural disagreement. Figure 1 illustrates the hinge points of motion that are identified for the region between residues 469 and 529 (Additional file 1: Figure S1 illustrates the results for the entire alignment). Residues corresponding to hinges are highlighted in the msTALI alignments shown in Table 3. Two points of motion for the thumb domain are identified by msTALI:residues G479 and A525 neatly delineate the 12° rotation of the H1 and H2 helices. The three structures exhibit a backbone RMSD of more than 5 Å over the backbone atoms of residues 479–525 after superimposing the remainder of the proteins. The high RMSD indicates the significant local structural change that has occurred over the three proteins. To validate the conservation of local structure, the RMSD of the same region can be obtained by superimposing only the local region (residues 479–525), as shown in Figure 2. This exercise yields an RMSD of less than 1.3 Å, which indicates the conservation of the local region as indicated by msTALI. Our analysis has also identified two additional hinge regions corresponding to residues T514 and A517. This region has been illustrated in gray in Figure 2. The original work also notes that the complete thumb domain undergoes motion, but the delineating residues are not identified by msTALI.

The points of rotation for the tip of the fingers domain, helices N and O, are identified as residues I638, the first residue in helix N, and L670, just after helix O. Two additional residues, P656 and L657, are also identified in the connecting loop as undergoing motion, as are two residues in the O1-O2 loop, A683 and P685. The delineating residue at the end of the fingers domain is not identified, but this is not unexpected, given the subtle nature of the motion involved. Residue A735 is also identified as a single point of structural alteration that does not correspond to any previous information reported in the literature.

### Comparison to previous methods

Our experiments have utilized two manually curated libraries of protein structures: Homstrad [29] and SABmark [30]. The popular Homstrad database is a manually curated set of 1032 multiple structure alignments, called *families*, each containing between 2 and 41 structures. A typical Homstrad family falls between the topology and homology levels of the CATH [31] database. To be consistent with previous analyses [12], we report our analysis for only the 399 families with at least three structures - that is, those families that constitute a multiple structure alignment. SABmark is a database of 425 families, each containing between 3 and 25 structures. Each family represents a SCOP superfamily. SABmark families are more divergent than Homstrad families and represent a more challenging test of the algorithm. We compare msTALI’s performance on Homstrad and SABmark databases to three of the most recognized multiple structure alignment programs - Mustang [10], POSA [11], and Matt [12]. It is important to note that all three comparing algorithms belong to the 3D class of structure determination algorithms while msTALI utilizes 1D linearization of structures. We chose to compare to 3D methods because they have proven more adept at identifying common cores [15,22]. Mustang is based on contact maps, small fragment alignments, and consensus-based methods. POSA and Matt both use aligned fragment pair chaining methods, and both allow backbone flexibility during the alignment process. All are sequential alignment methods, like msTALI. Mustang and Matt are compared on both Homstrad and SABmark, while POSA could only be compared on Homstrad. Homstrad and SABmark alignments for Matt were downloaded from the web at http://groups.csail.mit.edu/cb/matt/. Homstrad and SABmark alignments for Mustang were calculated locally after downloading the software from http://ww2.cs.mu.oz.au/~arun/Site/mustang.html. We computed RMSD and core size statistics ourselves for both applications to ensure consistency in calculation of RMSD scores.

Although we were unable to obtain results for POSA on SABmark, we nonetheless include Homstrad results due to POSA’s popularity and its emphasis on flexible structure comparison. POSA is not available for download, and so the statistics must be computed from available information. POSA outputs two structural alignments; one computed with bends disallowed, the other computed with bends allowed. Statistics for the unbent Homstrad alignments are available online at http://fatcat.burnham.org/POSA/POSAvsHOM.html. Statistics for the bent alignments are not available; the numbers from a previous analysis [12] are: core size 168, average RMSD 2.22 Å. POSA alignments for SABmark are not available.

We also demonstrate msTALI’s ability to create a protein core hierarchy using CATH domains. We extracted 341 domains and used them to construct a prototype library of protein core domains. We selected a subset of the CATH domains for analysis by randomly selecting 16 homologous superfamily levels and downloading all domains at the 35% sequence identity level. The chosen levels cover all four structural classifications (α, β, α-β, and mostly unstructured); within the α-helical class, we chose representatives that covered multiple categories for the architecture, topology, and homologous superfamily sublevels. Structures are compared using the core identification wrapper of msTALI, with its final score representing the distance between the core of two structures. Our final results were compared to the CATH classification with some interesting differences.

### Common core identification

Table 4 shows the performance of msTALI compared to Matt, Mustang, and POSA on the Homstrad database. This table shows the percentage of the 399 Homstrad families that msTALI outperforms its competitors on both core size and backbone RMSD; the percentage of families that msTALI does better on core size only; the percentage that msTALI does better on backbone RMSD only; and the percentage of families that the competitor does better on both measures. This detailed analysis shows that msTALI outperforms all competing applications by a significant margin.

**Table 4:** msTALI compared to Matt, Mustang, and POSA on 399 families from the Homstrad database

***msTALI* outperforms this program on**	**Matt**	**Mustang**	**POSA**
Core size and backbone RMSD	40.10%	57.5%	58.8%
Backbone RMSD	31.7%	18.7%	24.8%
Core size	22.2%	19.9%	13.2%
Neither	5.9%	3.8%	3.1%

msTALI finds *both* a larger core and smaller RMSD on 40.1%, 57.5%, and 58.8% of the families analyzed when compared to Matt, Mustang, and POSA respectively. This is true even when the training set families are removed (40.4%, 58.8%, and 57.5%, respectively). The results for most of the remaining families are ambiguous, with msTALI performing better on one measure but not another. However, the competing applications perform better than msTALI on both measures in only 5.9%, 3.8%, and 3.1% of the families, respectively.

**Figure 3:**
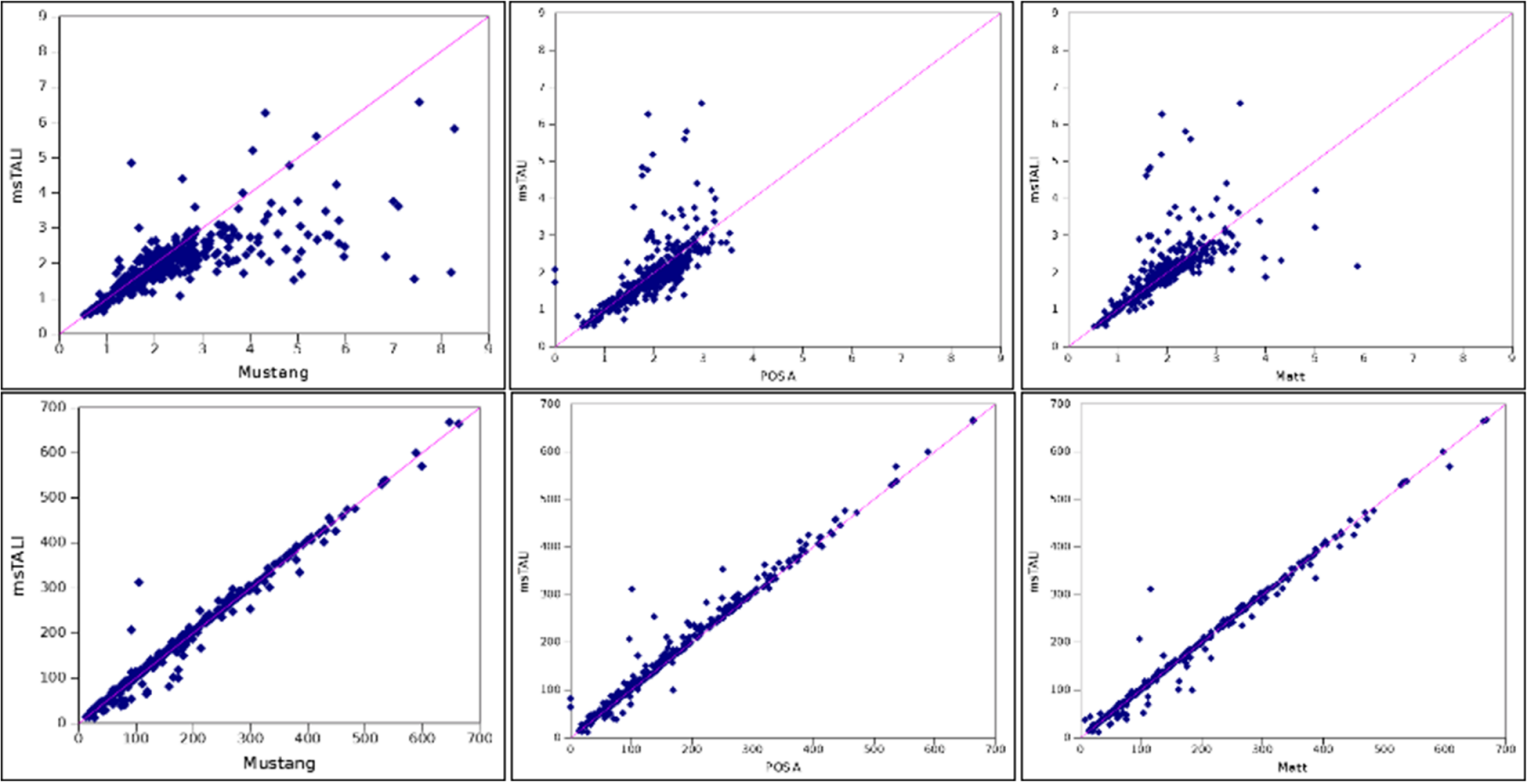
**Comparison of msTALI to competing algorithms on Homstrad and SABmark.** Comparison plots of the backbone RMSD (on top) and core size (on bottom) between msTALI and Mustang (left), POSA (middle), and Matt (right) on Homstrad. Backbone RMSD is measured in angstroms; core size is measured in residues. In all plots, msTALI is on the y-axis**.**

Figure 3 further illustrates msTALI’s performance on Homstrad. This figure plots backbone RMSD and core size for msTALI compared to Matt, Mustang, and POSA. The POSA core size plot is skewed well above the dividing line, clearly demonstrating that msTALI identifies many cores with larger sizes. Furthermore, a majority of the points in the RMSD plot lie below the line, illustrating the results from Table 4 that msTALI frequently locates protein cores with smaller RMSDs. The Mustang RMSD plot is skewed to the right; in particular, a number of Mustang cores have RMSDs higher than 7 Å, while msTALI has only a few. The core size plot is less conclusive; some core sizes are better for msTALI while others favor Mustang. The Matt RMSD plot is centered about the equality line, but the core size plot clearly shows that msTALI identified larger cores for a significant majority of the families. SABmark results on the 425 superfamily groups are shown in Table 5. POSA results are not available for SABmark, so the SABmark analysis includes only Matt and Mustang. msTALI exhibits excellent performance against Mustang, outperforming it on both core size and backbone RMSD for 43.6% of all groups. msTALI also outperforms Matt on 26.3% of all families. The results without the training families are 43.6% and 26.6% respectively. The competing applications perform better than msTALI on both measures in only 22.5% and 9.2% of the families, respectively. SABmark contains more challenging structure comparisons, and yet msTALI is able to achieve better results than the best competing algorithms.

**Table 5:** msTALI compared to Matt and Mustang on 425 families from the SABmark database

***msTALI* outperforms this program on**	**Matt**	**Mustang**
Core size and backbone RMSD	26.3%	43.6%
Backbone RMSD	27.0%	32.6%
Core size	24.3%	14.6%
Neither	22.5%	9.2%

**Figure 4:**
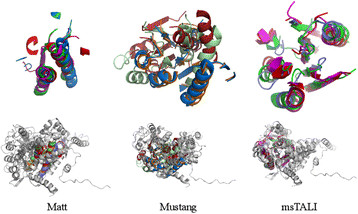
**Aligned structures from a Rossmann fold.** The conserved cores (top) and fully aligned structures (bottom) for the Rossmann fold family from SABmark, as aligned by Matt, Mustang, and msTALI. Only four structures are illustrated for clarity. Figures are rendered using PyMol [33]**.**

The data presented in Tables 4 and 5 and Figure 3 conclude that msTALI performs significantly better on the two comparison databases used for analysis. However, an example can be informative. Here we illustrate msTALI on a Rossmann fold [32] group from SABmark. The alignments produced by Mustang, Matt, and msTALI are shown in Figure 4. The core Rossmann fold is known to consist of a β-sheet of at least three strands enclosed by at least two α-helices. msTALI has correctly aligned the five central β-strands and the three surrounding α-helices. A fourth α-helix, in the top-right portion of the image, is partially conserved as well. In contrast, Matt has only aligned one β-strand and two α-helices. Mustang does somewhat better, aligning three β- sheets and two α-helices. However, it has also aligned α-helices from some structures with β-sheets of other structures. Furthermore, several of the secondary structures are not properly matched, resulting in a poor fit of the core between structures. The msTALI core contains 110 residues and a backbone RMSD of 2.2 Å. This is significantly better than cores identified by Matt, which has 54 residues and an RMSD of 3.5 Å, and Mustang, which has 110 residues and an RMSD of 4.5 Å. 

### Core structural phylogeny

The msTALI core and the core identification wrapper use a number of features from protein structures, such as surface accessibility, and a new approach to structure alignment, iterating from a general to a specific alignment. These changes necessitate a careful examination of the algorithm’s performance specifically related to reconstruction of the phylogenetic relationships. We examine the msTALI core in detail by using it to construct a phylogenetic tree. Constructing a phylogenetic tree of the relationships between structures requires a core that accurately reflects the relationships between two domains, regardless of how distantly they are related. This is a crucial step before performing multiple structure alignment. The phylogenetic tree is used to determine which structures are most similar and are aligned first.

We construct a phylogenetic tree using 341 CATH domains. This modest-sized sample provides sufficient data for insightful analysis but avoids the “analysis overload” of a large sample. To create the core library, we computed all pairwise distances between structures (using the msTALI score from the core identification wrapper), then used the results to construct a phylogenetic tree using the neighbor- joining algorithm [34].

We emphasize that it is not meaningful to directly compare CATH to a phylogeny tree constructed by msTALI. Our analysis focuses on identifying domains with similar cores, while CATH divides domains by class and allows order-independent comparisons at the architecture level. It is therefore not meaningful to perform a strict comparison between msTALI and CATH. However, we do expect that msTALI will cluster domains together at the topology and homologous superfamily levels as identified by CATH. In particular, the domains from a single superfamily class should be grouped together.

**Figure 5:**
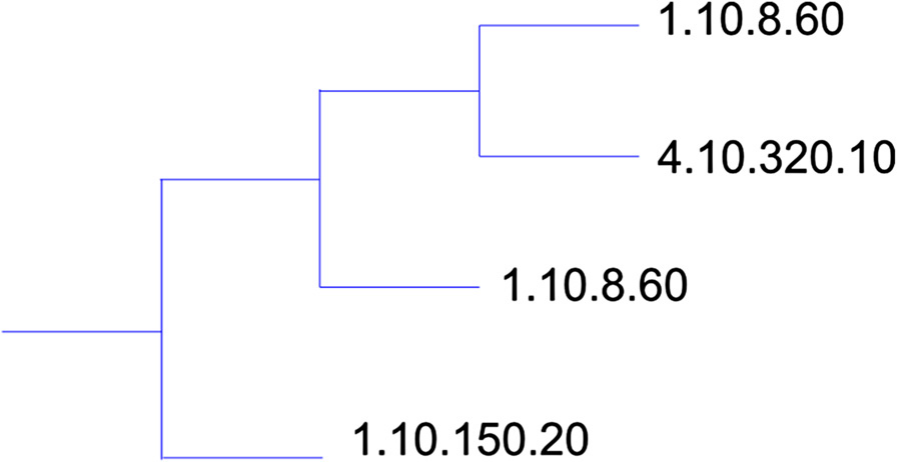
**A phylogenetic tree illustrating the division of the 1.10.8.60 CATH domains.** A phylogenetic tree for a portion of the CATH domains clustered with msTALI. This tree illustrates placement of the 4.10.320.10 cluster among the 1.10.8.60 clusters. The tree was rendered with TreeGraph 2 [35] and T-Rex [36]**.**

**Figure 6:**
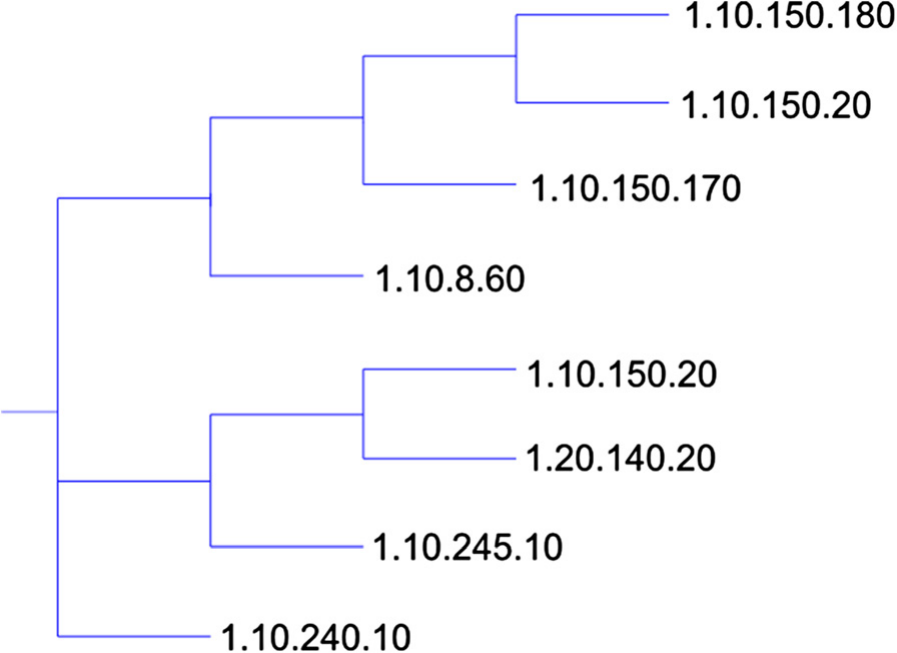
**A phylogenetic tree illustrating all α-helical CATH domains.** The compact phylogenetic tree for all α-helical CATH domains clustered with msTALI. The tree illustrates the splitting of the 1.10.150.20 superfamily into two clusters. The tree was rendered with TreeGraph 2 [35] and T-Rex [36]**.**

Our approach successfully placed each domain in the tree next to other domains from the same homologous superfamily. For the purposes of analysis, we define a *cluster* to be a subtree from our phylogeny tree that only contains domains from a single superfamily. We divided the tree into its maximal sized clusters. We expect that for most superfamilies, all of the superfamily’s domains will be contained in a single cluster. This is indeed the case; of the 16 superfamilies selected from CATH, 13 had all domains placed into a single cluster. Two superfamilies (1.10.8.60 and 1.10.150.20) were placed into two clusters; these divisions are illustrated in Figures 5 and 6. One superfamily (3.10.20.90) was placed into three clusters. The average cluster size was 17 domains, compared to the average CATH superfamily size of 21 domains. Domains in the three divided superfamilies were not evenly distributed among the multiple clusters. The largest cluster for 1.10.8.60 contained 94% of all domains from that superfamily, while the largest cluster for 1.10.150.20 contained 84% of all domains from that superfamily and 3.10.20.90 (not shown) contained 90% of the domains from that superfamily. While there are few differences with CATH at the homologous superfamily level, these differences warranted further investigation. There are two situations under which a superfamily might be divided into multiple clusters. The first is when all domains in a superfamily do not share a common core. The second is when domains from one superfamily have a core in common with another superfamily, and domains from the second superfamily divide the first superfamily into multiple clusters. We present an example of each situation from our results.

**Figure 7:**
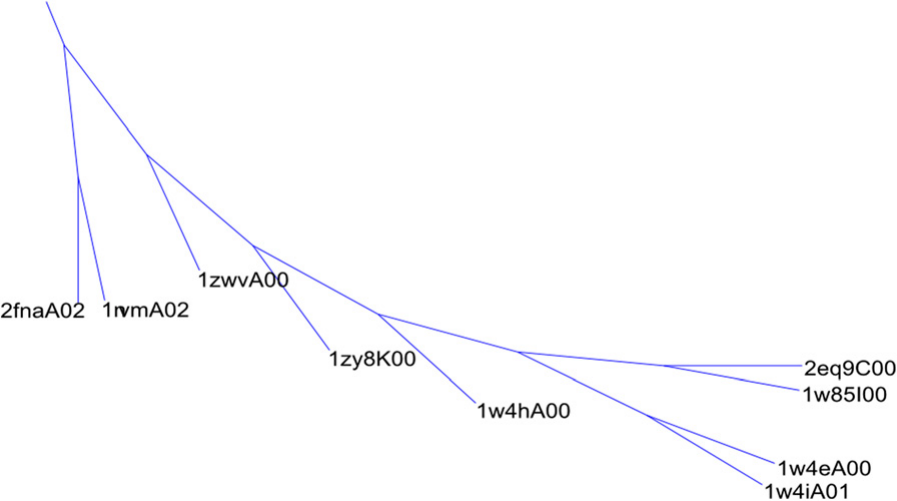
**A phylogenetic tree illustrating the CATH domains from 4.10.320.10 and 1.10.8.60.** A portion of the full phylogenetic tree. This subtree corresponds to the 4.10.320.10 cluster and the smaller 1.10.8.60 cluster. 2fnaA02 and 1nvmA02 are from 1.10.8.60; all other structures are from 4.10.320.10**.**

**Figure 8:**
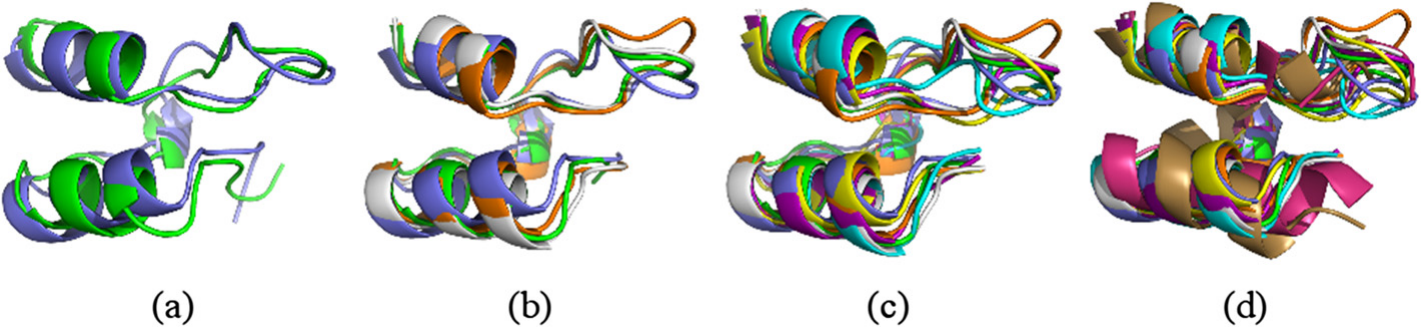
**An illustration of the protein cores derived from the CATH phylogenetic tree.** Protein cores extracted from the tree in Figure 6 by cutting the tree at various levels. The domains shown are: (**a**) 1w4e and 1w4i (**b**) those domains shown in (**a**) and 2 eq9 and 1w85 (**c**) those domains shown in (**b**) and 1w4h, 1zwv, and 1zy8 (**d**) those domains shown in (**c**) and 1nvm and 2fna. Groups (**a**) through (**c**) are from class 4.10.320.10, while the domains included in (**d**) are from domain 1.10.8.60**.**

**Figure 9:**
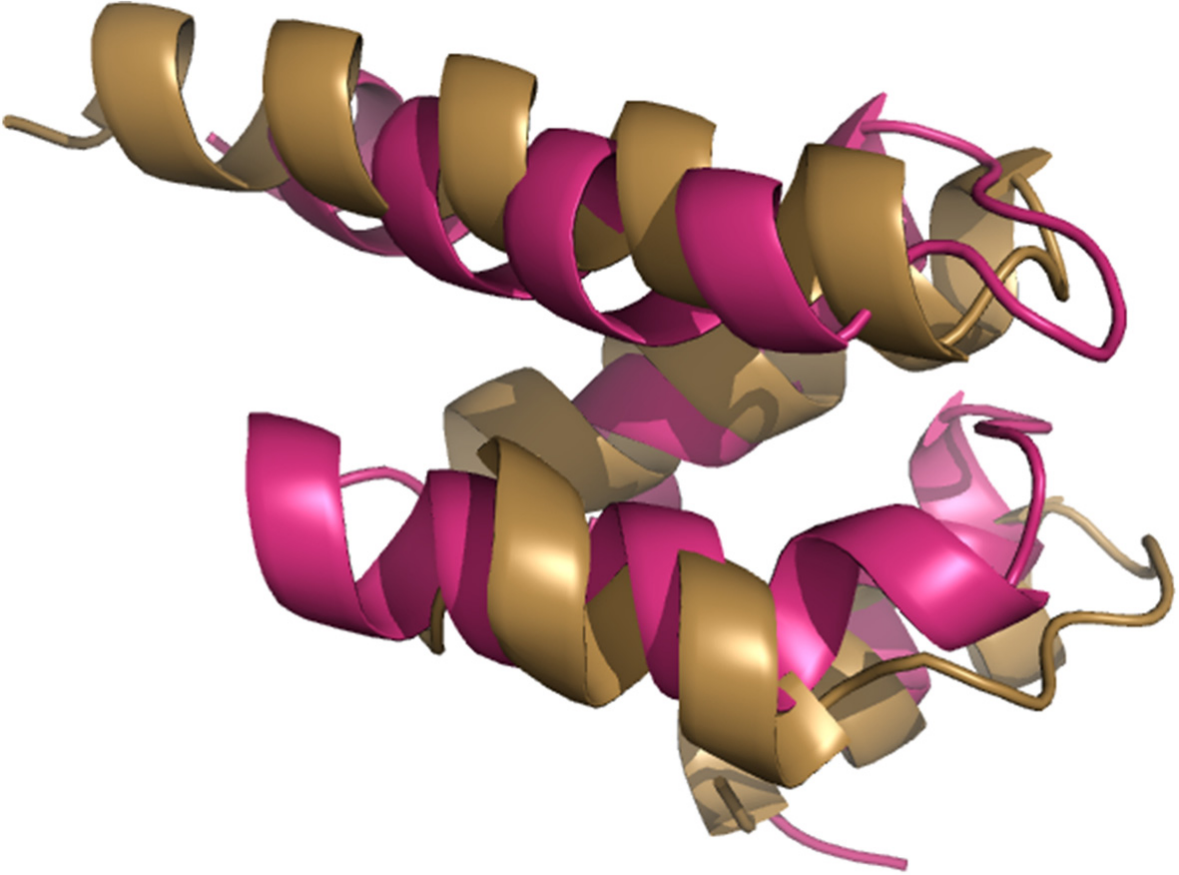
**A structural alignment of two domains from Figure**5**.** Two domains from Figure 6, CATH class 1.10.8.60, with the core identified and displayed separately.

The splitting of superfamily 1.10.8.60 occurs because the 4.10.320.10 domains have a strong core in common with those from 1.10.8.60, dividing 1.10.8.60 into two clusters. This splitting is illustrated in Figure 5. The larger 1.10.8.60 cluster contains 94% of that superfamily’s domains, and so deviations from CATH relate to the smaller 1.10.8.60 cluster. We examined the branches containing the 4.10.320.10 cluster and the smaller 1.10.8.60 cluster in more detail as shown in Figure 7. The protein cores created by cutting this portion of the phylogenetic tree at varying levels of similarity are shown in Figure 8. As more domains are incorporated into the core, some regions exhibit structural diversity, while others are nearly identical between domains. The regions of diversity are almost exclusively located in turns. It is remarkable that domains from the two classes contain substantial overlap between their cores. The core sizes are 42, 40, 39, and 40 residues for the cores labelled (a), (b), (c), and (d). The nine structures used to generate these cores range in size from 40 to 76 residues, with an average size of 50 residues. The common core size is 80% of the average domain size, lending substantial support to the conclusion that these structures from differing superfamilies are indeed built upon a single common core. Furthermore, while five of the seven domains are from 4.10.320.10, the two domains from 1.10.8.60 do not contain a core that is substantially larger than the common core displayed in Figure 8(d). The alignment of these two domains separately is shown in Figure 9.

The other reason a CATH superfamily could be divided into two or more clusters is that the domains in the superfamily do not share a single common core. This is the case for the domains from 1.10.150.20. These split domains are illustrated in Figure 6. One cluster contains 5 domains with an average size of 146 residues; the other cluster contains 27 domains with an average size of 66 residues. To examine the reason this class of homologous proteins was split into two clusters, we used msTALI to identify the cores for these two groups separately, and also for the groups when combined. These groups are denoted as follows: L for large group (i.e., the one with 27 domains), S for small group, and C for the combined group. Group L yielded three cores, with core sizes of 53, 56, and 41, and RMSDs of 3.49 Å, 2.63 Å, and 2.63 Å. Group S had a single core, with 82 residues and an RMSD of 2.9 Å. Group C yielded a single core with 15 residues and an RMSD of 2.70 Å. The core size for group C, the combined group, has too few residues to plausibly be considered as a structural “core” on which the domains are built. This is clear in light of the fact that the average domain size for group C is 79 residues. On the other hand, the cores for groups L and S comprise 76% and 56% of the average number of residues in each. From the stark difference in core size and RMSD between the combined group and the two groups identified by msTALI, it is clear that this division of the CATH class is necessary to yield meaningful protein cores. To confirm that this observed behavior is not an anomaly related to msTALI, we selected one domain from each of the two clusters - 2bcqA02 and 1tk5A04 - and aligned them using SSAP [37], one of the tools used in the construction of CATH. The two domains have 96% structural overlap (expressed as a percentage of the shorter domain), but it comes at the expense of an RMSD of 9.2 Å. Neither SSM [38] nor Matt was able to produce an alignment with a large core size and significantly lower RMSD (results not shown). We repeated this exercise for the remainder of the smaller cluster, using SSAP to compare each domain from the smaller cluster against a randomly selected domain from the larger cluster. The complete results are shown in Additional file 1: Table S5. The average number of residues in common was 96%, with an average RMSD of 8.6 Å.

## Conclusions

A flexible framework named msTALI is introduced in this report. msTALI can be customized to address a number of investigations centered around multiple structure alignment. msTALI achieves its broad potential by relying on an inclusive set of features that encapsulate a protein's structure and biochemistry. We have demonstrated that msTALI's linear representation of a structure combined with a dynamic programming algorithm, results in a fast and effective multiple structure alignment mechanism. The general framework that has been presented by msTALI can be of interest to a larger community of investigators. Through selection of various weight schemes and development of a relevant wrapper, the core msTALI algorithm can be extended to investigate a number of problems such as identification of active site or reconstruction of phylogenetic relationships. Using the customized core/wrapper combination, we demonstrated its success on several problems from the literature.

Analyzing the performance of msTALI on the reference alignments from Homstrad and SABmark has substantiated that msTALI's performance is very competitive compared to that of the most recent 3D methods such as MATT, POSA and Mustang. In particular, msTALI is effective in aligning structures from families where the structures vary widely in size. This is clear from the algorithm’s performance on SABmark, which contains many challenging families with structures of widely varying sizes. An example is illustrated in Figure 4, where the structures vary in size from 253 to 361 resides. Furthermore, we found that msTALI is frequently able to identify an alignment that includes fragments with larger cores without sacrificing the backbone RMSD. In particular, residues at the end of secondary structures or in loop regions were more often aligned correctly by msTALI (in comparison to the Homstrad alignment). These are critical residues that may be involved in functional activity of proteins and are often time missed during computational modeling of proteins.

msTALI is also novel compared to other approaches in that its starting point can be seeded based on results from other algorithms. Hybrid approaches can be easily implemented where msTALI's initial starting point is “seeded” based on results from another algorithm (such as Matt for example) in order to achieve an even higher performance than any one of these algorithms alone. For example, the seed alignment might come from an application that excels at aligning more divergent structures, or it might be manually constructed using *a priori* expert knowledge. Another example of seeding the initial condition of msTALI is in extraction of the conserved core motifs. We have demonstrated the success of the msTALI's internal mechanism of establishing structural relationships in order to guide the investigation of the conserved core motifs. It is entirely possible to confine the extraction of the core motifs to phylogenetic relationships other than the one that is internally calculated by msTALI. Here one can use relationships dictated by CATH or FSSP in order to obtain a different set of common cores.

We presented a phylogeny tree based on protein cores from CATH. The msTALI approach to phylogenetic reconstruction demonstrated strong similarity with the CATH classification with some noted differences. Such differences are common between standard tools (such as CATH, SCOP or FSSP for example) and further investigation of differences revealed strong evidence in favor of msTALI's classification of structures. Retrospectively, it should be expected that some α/β proteins or partially unstructured proteins will share common cores with α or β proteins. This type of differences are observed in classifications resulted from msTALI as shown in Figure 5.

Our future plans for msTALI include construction of a full core database from CATH domains. In addition, we expect that msTALI should perform well at identifying active sites from homologous proteins, whether using a pre-constructed database of homologous structures or by scanning the entire PDB for matches to a known query. Finally, we consider the PDB to be large enough that multiple structure alignments will become useful in many research areas, in addition to the ones presented.

## Implementation

We view the structure alignment problem as consisting of two related subproblems: *general* correspondence problem and *specific* correspondence problem. The general correspondence problem identifies corresponding fragments (typically secondary structures) between structures, but need not exactly align residues of the fragments. The specific correspondence problem is that of precisely aligning residues between structures to minimize backbone RMSD while maximizing the number of residues in the common core. Solving the general correspondence problem considerably simplifies the specific correspondence problem. The msTALI algorithm is designed to leverage this insight by computing a very effective initial alignment using information that provides an excellent general correspondence; it progresses to using properties that detect the exact residue-residue correspondences.

The algorithm contains two major components: a *core* and a *wrapper*. The core utilizes various features (such as torsion angles or surface accessibility) that are extracted from a set of structures in order to accomplish the task of general correspondence. The wrapper extends the capabilities of the algorithm by utilizing the results of the core in order to address a specific problem such as core identification or establishing phylogenetic relationships.

### msTALI core

The msTALI core is a sophisticated structure comparison algorithm. It is an extension of the previously reported TALI [20] algorithm in two major aspects. While TALI is based on torsion angles and sequence, msTALI includes additional structural and biochemical properties of the structures. msTALI also extends the core to allow alignment of multiple structures.

msTALI aligns two structures using a global dynamic programming algorithm and a linear representation of structures, in a manner similar to Needleman-Wunsch [39]. It uses an affine gap penalty with a gap opening penalty of 2.6 and a gap extension penalty of 0.4. The scoring function is shown in Eq. 1.

(1) $$\begin{array}{c} S(r_{i},r_{j})=w_{t}t(r_{i},r_{j})+w_{b}b(r_{i},r_{j})+w_{r}r(r_{i},r_{j})+w_{s}s(r_{i},r_{j})\\ +w_{\mathrm{dp}}d_{p}(r_{i},r_{j})+w_{\mathrm{sp}}s_{p}(r_{i},r_{j})+w_{\mathrm{ds}}d_{s}(r_{i},r_{j})+w_{\mathrm{ss}}s_{s}(r_{i},r_{j}) \end{array}$$

This scoring metric uses functions that compute the match between two residues *i* and *j*: *t* compares torsion angles, *b* compares global backbone C^α^ atom positions, *r* compares residue types, and *d*_*p*_*, d*_*s*_*, s*_*p.*_*,* and *s*_*s*_ compare the distance to and sequence types of neighboring residues. Each factor has a weight *w*_*−*_ and is collected into a weight vector ***w****;* the weights are normalized so that they sum to 1. The weights used in our analyses were determined from a small training set, which were used in allsubsequent analyses.

The torsion angle function *t* takes into account both torsion angles and secondary structure type. Torsion angles are compared using the transition cost through Ramachandran space as in TALI [20]. For a pair of torsion angles *(φ, ψ)*_*i*_, *(φ, ψ)*_*j*_ from residues ri and rj, the transition cost is t(ri, rj) = □∫R□l□dl, where L is the straight *L* path connecting points *(φ, ψ)*_*i*_, and *(φ, ψ)*_*j*_ and *R(p)* is the empirical log density at point *p* in Ramachandran space. Secondary structure types are determined using DSSP [40] and are compared using a fixed penalty for alignment between different types in order to mitigate the effect of aligning incompatible secondary structure types. This penalty was determined using a set of training structures.

The backbone atom function *b* compares backbone C^α^ positions using the Euclidean distance, with a maximum of 13 Å. The residue type comparison function *r* scores residues using BLOSUM30 [41]. The surface accessibility function *s* computes the absolute value of the difference in accessibilities and is useful in differentiating between buried and surface residues.

**Figure 10:**
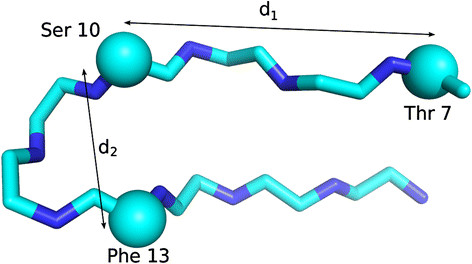
**An illustration of neighboring residues.** An illustration of neighboring residues from a portion of a protein structure. Cα atoms are shown as balls. For the residue in question, Ser 10, the N-terminal neighbor Thr 7 was identified, and its distance to Ser 10 d1 measured and sequence type s1 noted. Similarly, the C-terminal residue Phe 13 was identified and its distance d2 measured and sequence type s2 noted. While both neighboring residues are three residues from the target, this need not be the case**.**

Several properties of neighboring residues can play an important role in determining an overall alignment of structures. Several features related to relevant neighboring residues are therefore incorporated into the algorithm through the functions *d*_*p*_*, d*_*s*_*, s*_*p*_*,* and *s*_*s*_ to resolve potential ambiguities. An example is a series of antiparallel β-strands that form a β-sheet. If one strand is missing from a structure to be aligned, a flexible alignment algorithm may have difficulty identifying the correct correspondence between β-strands from different structures. We introduce *neighboring residues* to reduce this type of uncertainty. An example is shown in Figure 10. For any residue *i* (example Ser 10 in Figure 10), the closest preceding residue (in Euclidean distance measured between C^α^ atoms) among all preceding residues is identified, and the residue’s type *s*_1_ and the distance *d*_1_ are noted. To ensure that any of the two immediately preceding residues *i-1* or *i-2* is not always chosen, the chosen residue must be > 2 residues away in the primary sequence. This is repeated for the successive residues (*i +* *1* and *i +* *2* residues excluded), where the residue type is labeled *s*_2_ and the distance is labeled *d*_2_ . The comparison functions *d*_*p*_ and *d*_*s*_ each accept two residues numbered *i* and *j* from structures *m* and *n* and compute the differences in *d*_*1*_ and *d*_*2*_:

(2) $$d_{p}(r_{i,m},r_{j,n})=\left|d_{1}^{i,m}-d_{1}^{j,n}\right|$$

(3) $$d_{s}(r_{i,m},r_{j,n})=\left|d_{2}^{i,m}-d_{2}^{j,n}\right|$$

*s*_*p*_ and *s*_*s*_ compare the residue sequence types between preceding and succeeding residues using BLOSUM30:

(4) $$s_{p}(r_{i,m},r_{j,n})=\text{BLOSUM}30(s_{1}^{i,m},s_{1}^{j,n})$$

(5) $${\text{s}}_{\text{s}}(r_{i,m},r_{j,n})=\text{BLOSUM}30(s_{2}^{i,m},s_{2}^{j,n})$$

The corresponding weight for each factor in Eq. 1 is denoted by *w*. Furthermore, these weights may be specified separately for structured regions (α-helices and β- sheets) or turn regions. This allows the algorithm, for example, to adjust the contribution of the surface accessibility term. Because turn regions tend to be largely exposed, the accessibility is likely to be similar for all residues in a turn. In contrast, the surface accessibility in structured regions is reflective of the hydrophobic forces that affect folding and should be given greater emphasis. The sequence term is another that benefits from separate structured and turn weights. Some loops exhibit large structural variations between homologous structures, and emphasizing amino acid type to a greater degree can aid in successfully aligning these regions. Given the weights, comparing two residues consists of computing the weighted sum of all factors.

The residue-residue scores are in the range [0, 10] with 0 indicating the least similarity and 10 indicating the highest level of similarity. The score of a pairwise alignment is the average score over all aligned residues, and so it too lies in the range [0, 10]. Furthermore, an alignment score is independent of the order in which the structures are provided.

### Extension to multiple structure alignment

msTALI also extends the pairwise TALI to allow multiple structure alignment. It does so in a manner similar to ClustalW [3,42]. A phylogenetic tree containing all structures is computed using the neighbor-joining algorithm [34]. The tree is computed using scores from the pairwise core. The structures are then multiply aligned, using the tree as a guide. During the alignment, *profiles*[3,42] - sets of aligned structures - are used; the goal of the algorithm is to build more inclusive profiles until only a single one remains. Initially, each structure forms a profile of size 1 and is a leaf in the tree. The algorithm selects two profiles that share a common parent node and aligns them, then replaces the parent node with a node representing the aligned profile. This is performed repeatedly until a single profile remains.

Alignment of two profiles proceeds in the same manner as aligning two structures. When computing the score between two positions, each residue from one profile is compared to each residue from the other profile. In addition, each initial profile is weighted according to the branch length from the root to its corresponding leaf. These weights are applied to the residue-residue score in order to reduce the contribution of highly similar structures during the alignment process. The final score between two profile positions is then the weighted average over all pairwise scores.

During the course of multiple structure alignment, BB-RMSD is calculated over the backbone atoms of the entire alignment. In instances where some of the structures share alignment but other structures exhibit a gap, the BB-RMSD is calculated based on the available atomic coordinates. Therefore the contribution of the gapped regions to the overall BB-rmsd is limited to coordinates of the structures with available atomic coordinates.

### Core identification wrapper

**Figure 11:**
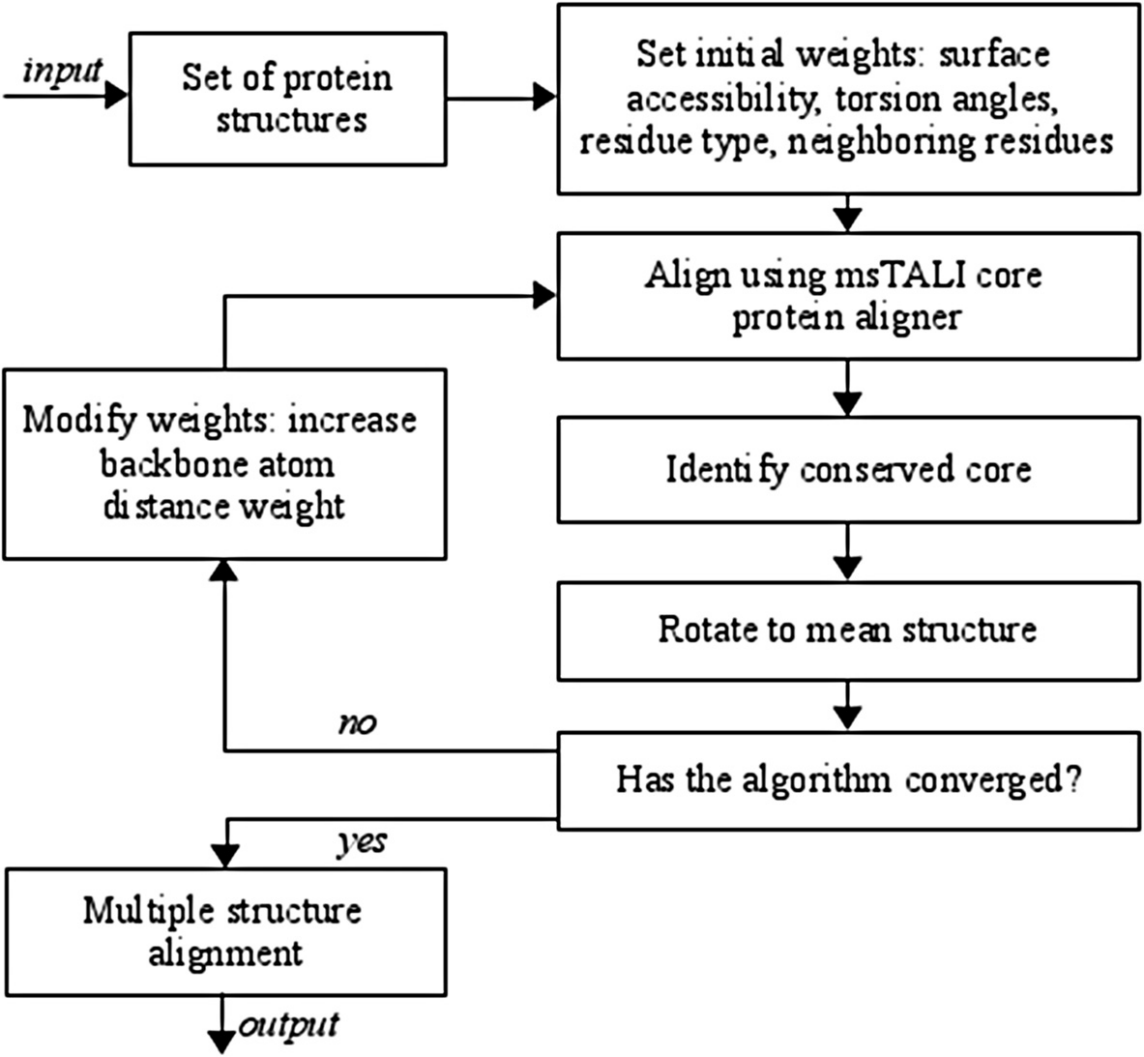
A flowchart of the msTALI algorithm.

The core identification wrapper is designed to extract a structural core from a set of homologous proteins. The criterion it optimizes is maximizing the number of residues included in the core with the constraint that the residues (potentially disjoint in sequence) fall under an RMSD cutoff. It is important to observe both of these criteria simultaneously since they are competing objectives. The complete algorithm is shown in Figure 11. This algorithm uses several parameters; the automated method for determining these parameters from a set of training examples.

The wrapper iteratively applies the msTALI core to progressively improve the multiple structure alignment. The first alignment computed by the wrapper is a general alignment and is performed using backbone torsion angles, sequence, surface accessibility, and the distance to, and amino acid types of, neighboring residues. It is reasonable to expect that some of these features (such as neighboring residues) will play an increasingly less important role in structure alignment. The weights of these features therefore diminish as the wrapper iterates and more emphasis is placed on C^α^ distances. The final iteration computes a specific alignment and uses only backbone atom distances. This, in effect, treats each structure as a rigid entity, allowing the algorithm to take into account the spatial orientations and geometry of various elements that constitute a folding pattern. These final iterations refine the specific correspondence between structures and enable msTALI to often include additional residues in the common core without increasing its backbone RMSD. The parameter determination method determines the initial and final weights. These are denoted ***w***_*I*_ and ***w***_*F*_, respectively. The weights used by the algorithm at iteration *i* are denoted ***w***_*i*_. The weights are updated after iteration *i* as ***w***_*i+1*_ = *0.5****w***_*i*_ + *0.5****w***_*F*_.

After each alignment is computed, *fragments*, ungapped stretches of residues, are identified. Fragments are merged into *motifs*, which are sets of fragments which yield a backbone RMSD under a user-defined threshold when considered together. This threshold is currently set to 2.5 Å, which corresponds to what experimental structural biologists consider as high structural significance. It is important to note that under this definition, some families in the test databases yield multiple motifs that cannot be merged together. From these motifs, the largest (in number of residues) is identified and defined as the main core of a group of proteins. Each structure is rotated to minimize the backbone RMSD over all core residues with respect to the first structure. The mean structure is computed over all residues in the core and each structure is rotated to minimize the backbone RMSD between it and the mean structure. Rotating to the mean structure moderates the effects of choosing a single structure as the reference structure. The mean structure can then be viewed as the evolutionary conserved core. After this process the wrapper updates the weights and decides whether or not to terminate. The algorithm iterates as long as the core size increases between iterations or the core RMSD decreases.

After the final alignment is computed, each position in the final profile is scored using only the backbone atom distance information. All residues with (1) scores greater than the experimentally determined cutoff threshold of 6.5 and (2) no gaps are considered part of the *conserved core*, which is the complete family core.

### Parameter determination

msTALI has several parameters that govern its behavior. These are primarily the weights for the various factors, but also include the motif merge threshold. To determine optimal values for all parameters in the core wrapper, ten families were extracted from Homstrad [29] and ten from SABmark [30]; these comprise less than 2.5% of the total database. The msTALI wrapper with hand-set parameters was used to align the families, and the final alignments were examined and confirmed to be satisfactory. All corresponding residue-residue pairs from the alignments were extracted and used to optimize the weights.

The secondary structure transition penalties were computed by observing the frequency of matches between secondary structure types and converting these values to expected probabilities. The values were scaled to be in the range [0, 10]. The match scores were computed for the torsion angle comparison function, and the difference from the expected probabilities computed. Linear regression was used to determine optimal penalties for the secondary structure transition penalties.

Weights for individual factors were set by extracting residue-residue pairs, treating them as ideal matches, and assigning each a target score of 10. A set of negative matches (of the same size) was generated by repeating the following: randomly select two non-paired residues, assume them to be an undesirable match, and assign them a target score of 0. Linear regression was then used to identify optimal weights. The weights for the algorithm’s initial iteration were set by eliminating the atom-atom distances, while the final weights were set by including these distances. The final weights had nearly zero values for all factors except the atom-atom distances, and so these values were zeroed.

The distance cutoff determines which residues are included in the conserved core. It sets the maximum allowed distance between a residue and the corresponding residue from the mean structure. This distance is computed as the Euclidean distance between C^α^ atoms. This cutoff controls the tradeoff between the number of residues belonging to the core and the average pairwise RMSD. Here the threshold was set using 20% of the Homstrad database. The appropriate threshold for a task ultimately depends on the user’s judgment as to whether a larger core size or a smaller RMSD is preferred.

Altation of msTALI's parameters provides a flexible means of customizing it for a specific task, therefore expanding the scope of msTALI's applicability. Additional file 1: Tables S6 and S7 list the parameters that are used for identification of core components of a structure, and flexible structure alignment respectively.

## **Availability and requirements**

**Project name:** msTALI

**Project home page:**http://ifestos.cse.sc.edu/wiki/html/index.php/MsTALI

**Operating system(s):** Linux

**Programming language:** C++

**Other requirements:** Dangle (available from http://kinemage.biochem.duke.edu/software/dangle.php) and DSSP [40] must be installed for standalone use

**License:** GNU GPL

**Any restrictions to use by non-academics:** licence needed

## Competing Interests

There are no competing interests related to this work.

## Authors’ contributions

PS designed the algorithm, wrote software, performed analyses, and wrote the paper. HV contributed to the algorithm, designed experiments and wrote the paper. All authors read and approved the final manuscript.

## Supplementary Material

Additional file 1 The C++ Source code for msTALI. The README file contains brief compilation and execution instructions. Click here for file

Additional file 2 **Table S1.** Full alignment of the mobile domains of DNA polymerase obtained from the STAMP analysis software. **Table S2**: Full alignment for mobile domains of DNA polymerase from MATT. **Table S3**: Full alignment for mobile domains of DNA polymerase from msTALI. **Table S4**: Configuration for study of Polymerase structures based on backbone torsion angles. All parameters not listed have zero values. **Table S5**: A comparison of the domains from 1.10.150.20 that were divided by msTALI into two separate clusters. The larger cluster contains 27 domains (84% of the total domains), while the smaller cluster contains 5 domains (16%). Each domain from the smaller cluster was compared to a randomly selected domain from the larger cluster using SSAP. Domains from the small cluster are on the left (Domain 1), while domains from the large cluster are on the right (Domain 2). These comparisons were performed to validate msTALI’s results, ensuring that this division was not related to an anomaly in msTALI. **Table S6**: Parameters of msTALI for core identification. All parameters not listed have zero values. **Table S7**: Parameters of msTALI for identification for flexible structure alignment. All parameters not listed have zero values. **Figure S1**: Per-residue score of msTALI for the three DNA polymerase proteins 1KTQ, 2KTQ and 3KTQ. Residues with scores more than 3s outside of the mean score were identified as the hinge regions. Click here for file
