# Supplementary material for: Multiple structure alignment with msTALI
Source: BMC Bioinformatics. 2012 May 20;13:105. doi: 10.1186/1471-2105-13-105 (PMC3473313; doi:10.1186/1471-2105-13-105)
Supplement: Additional file 2 — Table S1. Full alignment of the mobile domains of DNA polymerase obtained from the STAMP analysis software. Table S2: Full alignment for mobile domains of DNA polymerase from MATT. Table S3: Full alignment for mobile domains of DNA polymerase from msTALI. Table S4: Configuration for study of Polymerase structures based on backbone torsion angles. All parameters not listed have zero values. Table S5: A comparison of the domains from 1.10.150.20 that were divided by msTALI into two separate clusters. The larger cluster contains 27 domains (84% of the total domains), while the smaller cluster contains 5 domains (16%). Each domain from the smaller cluster was compared to a randomly selected domain from the larger cluster using SSAP. Domains from the small cluster are on the left (Domain 1), while domains from the large cluster are on the right (Domain 2). These comparisons were performed to validate msTALI’s results, ensuring that this division was not related to an anomaly in msTALI. Table S6: Parameters of msTALI for core identification. All parameters not listed have zero values. Table S7: Parameters of msTALI for identification for flexible structure alignment. All parameters not listed have zero values. Figure S1: Per-residue score of msTALI for the three DNA polymerase proteins 1KTQ, 2KTQ and 3KTQ. Residues with scores more than 3s outside of the mean score were identified as the hinge regions. [file 1471-2105-13-105-S2.pdf]

Supplementary Material

Table S1: Full alignment of the mobile domains of DNA polymerase obtained from the STAMP analysis software.

```
1KTQ     290 SPKALEEAPWPPPEGAFVGFVLSRKEPMWADLLALAAARGGRVHRAPEPYKALRDLKEAR 349
2KTQ     295 -----EEAPWPPPEGAFVGFVLSRKEPMWADLLALAAARGGRVHRAPEPYKALRDLKEAR 349
3KTQ     293 AL---EEAPWPPPEGAFVGFVLSRKEPMWADLLALAAARGGRVHRAPEPYKALRDLKEAR 349

1KTQ     350 GLLAKDLSVLALREGLGLPPGDDPMLLAYLLDPSNTTPEGVARRYGGEWTEEAGERAALS 409
2KTQ     350 GLLAKDLSVLALREGLGLPPGDDPMLLAYLLDPSNTTPEGVARRYGGEWTEEAGERAALS 409
3KTQ     350 GLLAKDLSVLALREGLGLPPGDDPMLLAYLLDPSNTTPEGVARRYGGEWTEEAGERAALS 409

1KTQ     410 ERLFANLWGRLEGEERLLWLYREVERPLSAVLAHMEATGVRLDVAYLRALSLEVAEEIAR 469
2KTQ     410 ERLFANLWGRLEGEERLLWLYREVERPLSAVLAHMEATGVRLDVAYLRALSLEVAEEIAR 469
3KTQ     410 ERLFANLWGRLEGEERLLWLYREVERPLSAVLAHMEATGVRLDVAYLRALSLEVAEEIAR 469

1KTQ     470 LEAEVFRLAGHPFNLNSRDQLERVLFDELGLPAISTS—A-----AV-LE--A---LREA  525
2KTQ     470 LEAEVFRLAGHPFNLNSRDQLERVLFDELGLP----AIGKTEKTGKRSTSAAVLEALREA 525
3KTQ     470 LEAEVFRLAGHPFNLNSRDQLERVLFDELGLP----AIGKTEKTGKRSTSAAVLEALREA 525

1KTQ     526 HPIVEKILQYRELTKLKSTYIDPLPDLIHPRTGRLHTRFNQTATATGRLSSSDPNLQNIP 585
2KTQ     526 HPIVEKILQYRELTKLKSTYIDPLPDLIHPRTGRLHTRFNQTATATGRLSSSDPNLQNIP 585
3KTQ     526 HPIVEKILQYRELTKLKSTYIDPLPDLIHPRTGRLHTRFNQTATATGRLSSSDPNLQNIP 585

1KTQ     586 VRTPLGQRIRRAFIAEEGWLLVALDYSQIELRVLAHLSGDENLIRVFQEGRDIHTETASW 645
2KTQ     586 VRTPLGQRIRRAFIAEEGWLLVALDYSQIELRVLAHLSGDENLIRVFQEGRDIHTETA-- 643
3KTQ     586 VRTPLGQRIRRAFIAEEGWLLVALDYSQIELRVLAHLSGDENLIRVFQEGRDIHTET--- 642

1KTQ     646 MFGVPREAVD-PL--M--R---------RAAKTINFG-VLYGMSAHRLSQEL-AIPYEEA 689
2KTQ     648 --S-----D--PL--M--R---------RAAKTINFG-VLYGMSAHRLSQEL-AIPYEEA 689
3KTQ     643 ----------ASWMFGVPREAVDPLMRRAA-KTINFGVL-YGMSAHRLSQELAIP-YEEA 689

1KTQ     690 QAFIERYFQSFPKVRAWIEKTLEEGRRRGYVETLFGRRRYVPDLEARVKSVREAAERMAF 749
2KTQ     690 QAFIERYFQSFPKVRAWIEKTLEEGRRRGYVETLFGRRRYVPDLEARVKSVREAAERMAF 749
3KTQ     690 QAFIERYFQSFPKVRAWIEKTLEEGRRRGYVETLFGRRRYVPDLEARVKSVREAAERMAF 749

1KTQ     750 NMPVQGTAADLMKLAMVKLFPRLEEMGARMLLQVHDELVLEAPKERAEAVARLAKEVMEG 809
2KTQ     750 NMPVQGTAADLMKLAMVKLFPRLEEMGARMLLQVHDELVLEAPKERAEAVARLAKEVMEG 809
3KTQ     750 NMPVQGTAADLMKLAMVKLFPRLEEMGARMLLQVHDELVLEAPKERAEAVARLAKEVMEG 809

1KTQ     810 VYPLAVPLEVEVGIGEDWLSAKE 832
2KTQ     810 VYPLAVPLEVEVGIGEDWLSAKE 832
3KTQ     810 VYPLAVPLEVEVGIGEDWLSAK- 831
```

Table S2: Full alignment for mobile domains of DNA polymerase from MATT.

```
1KTQ:A   SPKAL-EEAPWPPPEGAFVGFVLSRKEPMWADLLALAAARGGRVHRAPEPYKALRDLKEA   348  (A)
2KTQ:A   ------EEAPWPPPEGAFVGFVLSRKEPMWADLLALAAARGGRVHRAPEPYKALRDLKEA   348  (B)
3KTQ:A   -A---LEEAPWPPPEGAFVGFVLSRKEPMWADLLALAAARGGRVHRAPEPYKALRDLKEA   348  (C)

1KTQ:A   RGLLAKDLSVLALREGLGLPPGDDPMLLAYLLDPSNTTPEGVARRYGGEWTEEAGERAAL   408  (A)
2KTQ:A   RGLLAKDLSVLALREGLGLPPGDDPMLLAYLLDPSNTTPEGVARRYGGEWTEEAGERAAL   408  (B)
3KTQ:A   RGLLAKDLSVLALREGLGLPPGDDPMLLAYLLDPSNTTPEGVARRYGGEWTEEAGERAAL   408  (C)

1KTQ:A   SERLFANLWGRLEGEERLLWLYREVERPLSAVLAHMEATGVRLDVAYLRALSLEVAEEIA   468  (A)
2KTQ:A   SERLFANLWGRLEGEERLLWLYREVERPLSAVLAHMEATGVRLDVAYLRALSLEVAEEIA   468  (B)
3KTQ:A   SERLFANLWGRLEGEERLLWLYREVERPLSAVLAHMEATGVRLDVAYLRALSLEVAEEIA   468  (C)

1KTQ:A   RLEAEVFRLAGHPFNLNSRDQLERVLFDELGLPAIGKTEKTGKRS-------TSAA----   517  (A)
2KTQ:A   RLEAEVFRLAGHPFNLNSRDQLERVLFDELGLPA-----------IGKTEKTGKRSTSAA   517  (B)
3KTQ:A   RLEAEVFRLAGHPFNLNSRDQLERVLFDELGLPA-----------IGKTEKTGKRSTSAA   517  (C)

1KTQ:A   VLEALREAHPIVEKILQYRELTKLKSTYIDPLPDLIHPRTGRLHTRFNQTATATGRLSSS   577  (A)
2KTQ:A   VLEALREAHPIVEKILQYRELTKLKSTYIDPLPDLIHPRTGRLHTRFNQTATATGRLSSS   577  (B)
3KTQ:A   VLEALREAHPIVEKILQYRELTKLKSTYIDPLPDLIHPRTGRLHTRFNQTATATGRLSSS   577  (C)

1KTQ:A   DPNLQNIPVRTPLGQRIRRAFIAEEGWLLVALDYSQIELRVLAHLSGDENLIRVFQEGRD   637  (A)
2KTQ:A   DPNLQNIPVRTPLGQRIRRAFIAEEGWLLVALDYSQIELRVLAHLSGDENLIRVFQEGRD   637  (B)
3KTQ:A   DPNLQNIPVRTPLGQRIRRAFIAEEGWLLVALDYSQIELRVLAHLSGDENLIRVFQEGRD   637  (C)
```

```
1KTQ:A   IHTETASWMFGVPREAV---------------D-----PLMRRAAKTINFGVLYGMSAHR   677  (A)
2KTQ:A   IHTETAS----------WMFGVPREAV-----D-----PLMRRAAKTINFGVLYGMSAHR   677  (B)
3KTQ:A   IHTETAS--------------------WMFGVPREAVDPLMRRAAKTINFGVLYGMSAHR   677  (C)

1KTQ:A   LSQELAIPYEEAQAFIERYFQSFPKVRAWIEKTLEEGRRRGYVETLFGRRRYVPDLEARV   737  (A)
2KTQ:A   LSQELAIPYEEAQAFIERYFQSFPKVRAWIEKTLEEGRRRGYVETLFGRRRYVPDLEARV   737  (B)
3KTQ:A   LSQELAIPYEEAQAFIERYFQSFPKVRAWIEKTLEEGRRRGYVETLFGRRRYVPDLEARV   737  (C)

1KTQ:A   KSVREAAERMAFNMPVQGTAADLMKLAMVKLFPRLEEMGARMLLQVHDELVLEAPKERAE   797  (A)
2KTQ:A   KSVREAAERMAFNMPVQGTAADLMKLAMVKLFPRLEEMGARMLLQVHDELVLEAPKERAE   797  (B)
3KTQ:A   KSVREAAERMAFNMPVQGTAADLMKLAMVKLFPRLEEMGARMLLQVHDELVLEAPKERAE   797  (C)

1KTQ:A   AVARLAKEVMEGVYPLAVPLEVEVGIGEDWLSAKE--   832  (A)
2KTQ:A   AVARLAKEVMEGVYPLAVPLEVEVGIGEDWLSAK-E-   832  (B)
3KTQ:A   AVARLAKEVMEGVYPLAVPLEVEVGIGEDWLSAK--E   832  (C)
```

Table S3: Full alignment for mobile domains of DNA polymerase from msTALI.

```
                                           450
1KTQ erlfanlwgrlegeerllwlyreverplsavlahmeatgvrldvaylralslevaeeiar
2KTQ erlfanlwgrlegeerllwlyreverplsavlahmeatgvrldvaylralslevaeeiar
3KTQ erlfanlwgrlegeerllwlyreverplsavlahmeatgvrldvaylralslevaeeiar
                                                ------H----------


            480                                         520
1KTQ leaevfrlaghpfnlnsrdqlervlfdelglpai---------stsaavlealreahpiv
2KTQ leaevfrlaghpfnlnsrdqlervlfdelglpaigktektgkrstsaavlealreahpiv
3KTQ leaevfrlaghpfnlnsrdqlervlfdelglpaigktektgkrstsaavlealreahpiv
     ---------         -H1-----                   -H2---      --

                                 560
1KTQ ekilqyreltklkstyidplpdlihprtgrlhtrfnqtatatgrlsssdpnlqnipvrtp
2KTQ ekilqyreltklkstyidplpdlihprtgrlhtrfnqtatatgrlsssdpnlqnipvrtp
3KTQ ekilqyreltklkstyidplpdlihprtgrlhtrfnqtatatgrlsssdpnlqnipvrtp
     ----I------------------      ---7---      ---8---          -

             600                                        640
1KTQ lgqrirrafiaeegwllvaldysqielrvlahlsgdenlirvfqegrdihtetaswmfgv
2KTQ lgqrirrafiaeegwllvaldysqielrvlahlsgdenlirvfq-grdihtetas-----
3KTQ lgqrirrafiaeegwllvaldysqielrvlahlsgdenlirvfqegrdihtetaswmfgv
     ---K-----     ----9--- ---L-------  ---M-----   ----N----

                                 680
1KTQ preavdplmrraaktinfgvlygmsahrlsqelaipyeeaqafieryfqsfpkvrawiek
2KTQ -----dplmrraaktinfgvlygmsahrlsqelaipyeeaqafieryfqsfpkvrawiek
3KTQ preavdplmrraaktinfgvlygmsahrlsqelaipyeeaqafieryfqsfpkvrawiek
             ----O-------    --O1--      --O2----------    ------

   710                           740
1KTQ tleegrrrgyvetlfgrrryvpdlearvksvreaaermafnmpvqgtaadlmklamvklf
2KTQ tleegrrrgyvetlfgrrryvpdlearvksvreaaermafnmpvqgtaadlmklamvklf
3KTQ tleegrrrgyvetlfgrrryvpdlearvksvreaaermafnmpvqgtaadlmklamvklf
     -P------ -10     -11         -------Q------------------------


   770
1KTQ prleemgarmllqvhdelvleapkeraeavarlakevmegvyplavplevevgigedwls
2KTQ prleemgarmllqvhdelvleapkeraeavarlakevmegvyplavplevevgigedwls
3KTQ prleemgarmllqvhdelvleapkeraeavarlakevmegvyplavplevevgigedwls
     ----           ---13---   --R----------          --14---

1KTQ ake
2KTQ ake
3KTQ ak-
```

Table S4: Configuration for study of Polymerase structures based on backbone torsion angles. All parameters not listed have zero values.

Section	Parameters
General	gapDistanceWeight=0.4<br><br>gapOpen=6.0<br><br>gapExtension=1.0
SSParams	sequenceWeight=1.0
TurnParams	sequenceWeight=1.0
SSParamsFinal	angleWeight=1.0
TurnParamsFinal	angleWeight=1.0

Table S5: A comparison of the domains from 1.10.150.20 that were divided by msTALI into two separate clusters. The larger cluster contains 27 domains (84% of the total domains), while the smaller cluster contains 5 domains (16%). Each domain from the smaller cluster was compared to a randomly selected domain from the larger cluster using SSAP. Domains from the small cluster are on the left (*Domain 1*), while domains from the large cluster are on the right (*Domain 2*). These comparisons were performed to validate msTALI's results, ensuring that this division was not related to an anomaly in msTALI.

Domain 1	*Size (residues)*	*Domain 2*	*Size (residues)*	*Backbone RMSD (Å)*	*Core size (residues)*	*SSAP score*
1aroP05	208	1u91B00	70	4.88	70	57.61
2ktqA04	133	1dgsA05	69	9.16	67	54.61
2kfnA04	144	1cukA02	76	7.04	71	61
2hhvA04	144	1lb2B00	72	12.75	65	53.93
1tk5A04	99	2bcqA02	54	9.2	52	60.02

Table S6: Parameters of msTALI for core identification. All parameters not listed have zero values.

Section	Parameters
General	gapDistanceWeight=0.4<br>gapOpen=6.0<br>gapExtension=1.0<br>iterate: true<br>realign: true
Params	g2DistWeight = -0.1331<br>g2SeqWeight = 0.2642<br>l2DistWeight = 0.0885<br>l2SeqWeight = 0.2443
SSParams	angleWeight=0.5231<br>sequenceWeight=0.4795<br>surfaceAccessibilityWeight=-0.2210
TurnParams	angleWeight=-0.2268<br>sequenceWeight=0.6889<br>surfaceAccessibilityWeight=-0.0787
SSParamsFinal	distanceWeight=1.0
TurnParamsFinal	distanceWeight=1.0

Table S7: Parameters of msTALI for identification for flexible structure alignment. All parameters not listed have zero values.

Section	Parameters
General	endGapExtension=1.0<br>gapDistanceWeight=0.4<br>gapOpen=6.0<br>gapExtension=1.0
SSParams	angleWeight=0.6<br>sequenceWeight=0.4
TurnParams	angleWeight=0.6<br>sequenceWeight=0.4
SSParamsFinal	angleWeight=0.6<br>sequenceWeight=0.4
TurnParamsFinal	angleWeight=0.6<br>sequenceWeight=0.4

Figure S1: Per-residue score of msTALI for the three DNA polymerase proteins 1KTQ, 2KTQ and 3KTQ. Residues with scores more than 3s outside of the mean score were identified as the hinge regions.
